# A new point-of-care test for the rapid antimicrobial susceptibility assessment of uropathogens

**DOI:** 10.1371/journal.pone.0284746

**Published:** 2023-07-05

**Authors:** Alyexandra Arienzo, Lorenza Murgia, Valentina Cellitti, Valeria Ferrante, Ottavia Stalio, Francesca Losito, Valentina Gallo, Federica Tomassetti, Rossella Marino, Flavia Cristofano, Michela Orrù, Paolo Visca, Salvatore Di Somma, Lorena Silvestri, Vincenzo Ziparo, Giovanni Antonini

**Affiliations:** 1 Interuniversity Consortium “Biostructures and Biosystems National Institute” (INBB), Rome, Italy; 2 Department of Science, Roma Tre University, Rome, Italy; 3 MBS srl, Rome, Italy; 4 Emergency Medicine, Department of Medical-Surgery Sciences and Translational Medicine, Sant’Andrea Hospital, Sapienza University of Rome, Rome, Italy; 5 Istituto Dermopatico dell’Immacolata, Rome, Italy; University of Rome Tor Vergata: Universita degli Studi di Roma Tor Vergata, ITALY

## Abstract

Bacterial resistance to antimicrobials is considered a major issue worldwide. This condition may account for treatment failure of urinary tract infections, which are among the most common infections both in community and healthcare settings. Therapy against uropathogens is generally administered empirically, possibly leading to unsuccessful therapy, recurrence and development of antibiotic resistance. The reduction in analytical time to obtain antimicrobial susceptibility test (AST) results could play a key role in reducing the cost of healthcare, providing information about antibiotic efficacy and thus preventing from either exploiting new and expensive antibiotics unnecessarily or using obsolete and ineffective ones. A more rational choice among treatment options would hence lead to more effective treatment and faster resolution. In this paper we evaluated the performance of a new Point Of Care Test (POCT) for the rapid prediction of antimicrobial susceptibility in urine samples performed without the need of a laboratory or specialized technicians. 349 patients were enrolled in two open-label, monocentric, non-interventional clinical trials in partnership with an Emergency Medicine ward and the Day Hospital of two large healthcare facilities in Rome. Antibiogram was carried out on 97 patients. Results from analysis of urine samples with the POCT were compared with those from routine AST performed on culture-positive samples, displaying high accuracy (>90%) for all tested antimicrobial drugs and yielding reliable results in less than 12 hours from urine collection thus reducing analytical and management costs.

## Introduction

Urinary tract infections (UTIs) are among the most prevalent infectious diseases in both community and healthcare settings [1, 2]. Their prevalence is higher in women than in men [3], due to several factors such as hormonal changes, sexual activity, and, above all, anatomical structure, with an average 30–60% prevalence in women, that varies depending on the age group [4]. The most common causative agent is *Escherichia coli*, that accounts for about 80% cases in community setting, followed by other *Enterobacteriaceae* and *Staphylococcaceae* [5, 6].

Among hospital settings, UTIs account for almost 40% of all nosocomial infections, and represent a major burden, given the associated morbidity and mortality. A correlation between urinary tract infections and systemic diseases has been recently suggested as patients displaying bacteriuria due to drug-resistant pathogens are more prone to develop sepsis, often due to treatment failure [7].

The emergence of antimicrobial resistance (AMR) is a major public health challenge that is threatening the effective prevention and treatment of an ever-increasing range of infections. Mortality rates associated to drug-resistant microorganisms are expected to rise in the next decades. As stated by the National Institute for Health and Care Excellence (NICE), by 30 years up to 10 million people could be under death threat due to unsuccessful antibiotic treatment [8].

Uropathogens vary from the community to the hospital setting, with different main agents prevailing and higher AMR frequencies in the latter; this causes a greater difficulty to achieve successful treatment, associated with longer hospitalization, patient’s uneasiness and overall higher costs [9–11].

Suspected UTIs are commonly treated with antibiotics, and guidelines recommend starting an empirical treatment before the results of urine culture and AST are available [12–14]. Empirical antimicrobial regimens should be based on local resistance patterns, and take into account the continually changing rates of antimicrobial resistance [14–21]. This is however, not always applicable, given the great local variability, undermining the outcome of empiric therapy [22, 23].

In this context, the importance of using evidence-based strategies for the treatment of UTI becomes undeniable: a fast and accurate diagnosis, leading to a rational treatment, is in fact essential to provide a timely and successful therapy.

The National Institute of Clinical Excellence has long been demanding the improvement and implementation of urine testing seeking for markers of bacterial infection in order to avoid empiric treatment for suspected UTIs [24]. Primary care accounts for most of antibiotic prescriptions implying that strict control should be applied to this setting for a more rational antibiotic usage [25].

In this context, Point of care Tests (POCTs) for UTI diagnosis, where testing is provided close to or near the patient, are currently being studied; their implementation in routine practice could facilitate the screening of UTIs, allow prompt treatment only when needed, and ultimately optimize workflows in order to reduce the burden on central laboratories, allow adherence to guidelines and improve therapeutical outcomes [26–29].

In a previous paper we described a new POCT for UTI detection upon culture of urine samples based on the Micro Biological Survey method. This POCT demonstrated to be accurate and time saving: performed in ready to use, disposable reaction vials and based on the time taken for the reaction vials to change color. It allows a quantitative assessment in of viable bacteria concentration in urine samples in less than 5 hours, not needing other instrumentation than a dedicated thermostatic reader which automatically provides results, opening for automation and processing of multiple samples [30, 31]. The same methodology was implemented in a new POCT for antibiotic susceptibility testing, developed by MBS Diagnostics ltd (London, UK), which is the focus of this work.

Here, the proficiency of the MBS AST-POCT was tested in two trials conducted between 2015 and 2017 in two clinical Departments differing in demographic and clinical profile of cared patients, in order to achieve a broader and realistic assessment of the performances of the AST-POCT in both community and hospital settings.

## Material and methods

### Study design

A total of 349 patients were enrolled in two open-label, monocentric, non-interventional clinical trials in collaboration with the Department of Emergency Medicine at Azienda Ospedaliera Sant’Andrea, Rome and the outpatient clinic at Istituto Dermopatico dell’Immacolata, Rome; 101 and 248 patients were enrolled in the two facilities, respectively, between November 2015 and December 2017. Prior to study participation, each patient was asked to read carefully through the patient information sheet and signed the informed consent. Approval of the study was obtained on 14.01.2013 from the Ethical Committee of Azienda Ospedaliera Sant’Andrea, and on 25.05.2017 from the Ethical Committee of Istituto Dermopatico dell’Immacolata, constituted according to DM 12.05.2006 following Good Clinical Practice. Authorization was given based on the declaration that the patients were duly informed and consenting. Only patients subscribing the informed consent were enrolled. The clinical trial did not imply any change in the normal diagnostic and therapeutic procedures.

Among patients enrolled in the two studies, AST was carried out on a total of 97 patients diagnosed with UTI upon urine culture, after admission in the health care facilities.

### First clinical trial: Azienda Ospedaliera Sant’Andrea, Rome

In the first clinical trial performed at the Department of Emergency Medicine at Azienda Ospedaliera Sant’Andrea, Rome, analyses were conducted on urine samples immediately after collection, in parallel with the MBS AST-POCT and the reference culture method for bacterial load assessment, and AST, performed by the hospital clinical microbiology laboratory.

During this trial the impact of urine samples freezing on MBS AST-POCT results was also assessed.

### Urine collection

Urine samples were provided by the hospital staff after collection in the morning. Midstream urine (MSU) samples or catheter specimens were collected using disposable, sterile containers, kept refrigerated for maximum 2 hours and split into 10 aliquots of 1 ml each: 4 were immediately analyzed by the hospital laboratory and with the MBS AST POCT, 3 were frozen at -80°C using sterile glycerol at a final 15% (vol/vol) concentration, while the other 3 were further divided in 100 μl aliquots, frozen at -80°C using sterile glycerol at a final 15% (vol/vol) concentration, and analyzed within 7 days, to assess the impact of sample freezing or in case of the need of further verification analysis.

### Hospital laboratory tests

Urine culture was the reference method used for confirmation of UTI suspicion. Bacterial identification and AST were performed using the VITEK^®^ MS and VITEK^®^ 2 systems (BioMérieux Italia S.p.a., Florence, Italy) with 64-well cartridges for AST, according to the manufacturer’s recommendations. Results were provided by the hospital laboratory in maximum 7 days.

### Antimicrobial Susceptibility Testing using the AST-POCT

The MBS AST-POCT analysis is performed in ready to use disposable vials containing the lyophilized growth medium used for the detection of UTI [31] alone or supplemented with selected antibiotics, namely amoxicillin, ciprofloxacin, trimethoprim-sulfamethoxazole and levofloxacin, chosen as first-line treatment options according to hospital guidelines. Antibiotics are added at clinical breakpoint concentrations according to European Committee on Antimicrobial Susceptibility Testing [32]: 8 μg/ml for amoxicillin-clavulanic acid, 1 μg/ml for ciprofloxacin, 4 μg/ml for trimethoprim-sulfamethoxazole, 1 μg/ml for levofloxacin.

For the MBS AST-POCT analysis, urine samples were tested in parallel using antibiotic-free and antibiotic-supplemented vials. Samples (20 μl) were manually injected in the rehydrated vials (final volume 10 ml) and incubated in a dedicated thermostatic optical reader, the MBS multireader, at 37°C for 26 hours. At the end of the analysis, vials were sterilized with sodium dichloroisocyanurate by pressing their cap and then were disposed of as “Non-Hazardous Waste” [31, 33].

The MBS AST-POCT is a colorimetric system that allows a semi-quantitative assessment of viable bacteria concentration. Different from other culture-based methods, it measures the enzymatic activity associated with bacterial metabolism, allowing results to be obtained in short time. Vials change color from blue to yellow in times that are inversely related to the bacterial concentration in the sample.

The AST POCT results were obtained by comparing the time taken by the antibiotic-free vial and the antibiotic-supplemented vials to change color. Regarding antibiotic free vials, bacteriuria, i.e. presence of ≥ 10^5^ colony-forming units (CFU)/ml, is detected upon blue to yellow color change of the medium in the reaction vial within 12 hours. Regarding antibiotic supplemented vials a color change from blue to yellow of the reaction vials within 12 hours, i.e., a positive result, indicates that the infecting bacterial metabolism and growth is not impaired by the presence of the antibiotic; on the other hand, an absence of color change or a significant delay of the time for color change, i.e. a negative result, indicates that the infecting bacterial metabolism and growth is significantly impaired by the presence of the selected antibiotic. In case of confirmed UTI (color change of the antibiotic-free vial within 12 hours) the infecting bacteria were predicted to be antibiotic-susceptible if no color change was observed in the antibiotic supplemented vials (denoting complete inhibition), or if the time taken for the antibiotic supplemented vials was considerably longer than that recorded for the vial without antibiotics. Bacteria were instead predicted to be resistant if the time for color change of the antibiotic-free vial and the antibiotic-supplemented vials antibiotic were comparable.

The vials used in the study were provided by MBS Diagnostics ltd (London, UK) and produced by Sclavo Diagnostics spa (Sovicille, SI, Italy) in compliance with requirements set in the IVDD (EU In Vitro Diagnostic Directive) and following the Quality Control System confirmed by ISO 9001 and ISO 13485 certifications. Five independent production batches of vials were used throughout the two trials.

### Evaluation of the impact of sample freezing on MBS AST-POCT results

In order to assess the impact of freezing MBS AST-POCT results, analyses were performed only on frozen culture-positive samples analyses, i.e., on samples for which urine culture and AST results were made available by the hospital’s laboratory. Analyses were performed on a total of 10 urine samples. For each sample aliquots (100 μl each) were stored at -80°C and stored up to 7 days. Analyses were performed in duplicate on each sample after 2, 5 and 7 days from collection at the Science Department of Roma Tre University, Rome. Considering the dilution factor, due to the addition of glycerol, 40 μl of the frozen aliquots were analyzed with MBS AST-POCT and results were compared with those previously obtained for the same fresh samples.

### Second clinical trial: Istituto Dermopatico dell’Immacolata, Rome

In the second trial, at Istituto Dermopatico dell’Immacolata, the MBS AST-POCT analyses were performed only on culture-positive samples, i.e., on samples for which urine culture and AST results were made available by the hospital’s laboratory. Analyses were performed on frozen samples, allowing the possibility to expand the pool pf patients tested and to obtain statistically significant results.

### Urine collection

Urine samples were collected by medical staff in the morning in the outpatient clinic or provided by patients themselves following self-sampling of first morning midstream clean-catch urine specimens. Samples after collection were kept refrigerated for maximum 2 hours and split into 10 aliquots of 1 ml each: 5 were sent to the hospital laboratory while the other 5 were frozen at -80°C using sterile glycerol at a final 15% (vol/vol) concentration. The latter samples were used for antibiotic susceptibility assessment with the MBS AST-POCT after central laboratory results were available, and in all cases within 48 hours. Supplementary aliquots were stored at -80°C in case of the need of further verification analysis.

### Hospital laboratory tests

Urine culture, bacterial identification and AST were performed as previously described for the first clinical trial.

### Antimicrobial Susceptibility Testing using the AST-POCT

The MBS AST-POCT analyses were carried out not later than 48 hours from collection, on frozen aliquots of urine samples. This allowed to perform analyses only on patients confirmed as positive by the hospital laboratory after urine culture, and consequently undergoing antibiotic susceptibility analysis, and not on every patient received at the hospital. Analyses were performed as previously described.

### Ex-post verification analysis

In case of discordance between the results obtained with the reference method, performed by the central laboratory within the hospital, and the MBS AST POCT, a verification analysis was performed at the Science Department of Roma Tre University, Rome, strictly within one or two days, to investigate the source of discordance. Analyses were repeated with both reference method and MBS AST POCT using aliquots of urine samples stored at -80°C using sterile glycerol at a final 15% (vol/vol) concentration maximum after 7 days from collection. The reference method chosen for ex-post analysis was the Kirby-Bauer diffusion disk test, as the traditional and most widely used approach [34]. Urine samples were plated onto non-selective solid media, such as Muller-Hinton agar (Liofilchem, Roseto degli Abbruzzi, TE, Italy), and a sterile disk containing a standardized concentration of the antibiotic of interest was positioned on the plate using sterile forceps. A negative control (blank disk without antibiotic) was also placed on the agar surface. After 24 hours incubation at 37°C, the possible presence of a halo of inhibition surrounding the disk containing the antibiotic was recorded; the diameter of halos was measured and confronted with standard tables to define resistance and susceptibility conditions. Analysis with the MBS AST POCT were performed according to the protocol described above (“*Antimicrobial Susceptibility Testing using the AST-POCT”*). In case of concordance between the results obtained with both methods, no further analysis was performed and data were used to carry out statistical analysis. In case of discordance, two possible scenarios were considered: *i)* when the MBS AST POCT confirmed a positive result (meaning bacterial resistance) and verification method showed a halo of inhibition 8meaning bacterial susceptibility), the MBS AST POCT results were definitively considered false positive; *ii*) when the MBS AST POCT confirmed a negative result (meaning bacterial susceptibility) and no zones of inhibition were detected through Kirby-Bauer technique (meaning bacterial resistance), results were definitively considered false negative.

## Results and discussion

The MBS AST POCT is a device with a simple operating protocol that can be used by healthcare staff at the patient’s bedside, and has been developed with the aim to provide fast and reliable information concerning the effectiveness of first-line antibiotics, thus helping clinicians choose the best treatment.

Standard AST provides results in at least 48 hours, and, for this reason, antibiotic therapy following UTI diagnosis is usually started on empirical basis, possibly leading to incorrect treatment and ultimately participating in the rise in bacterial antibiotic resistance. In order to improve treatment, providing a more rational and effective antibiotic administration, a reduction in analytical time is desirable, allowing medical staff to base therapy on evidence-based information. This would prevent from either exploiting new and expensive antibiotics unnecessarily or using ineffective ones and lead to more effective treatment and faster resolution.

In this work, the accuracy of antimicrobial susceptibility assessment using the MBS AST POCT was investigated. Two different clinical settings were taken into account, both a first-aid and an outpatient clinical framework, reflecting two very different patient pools.

### Patients’ characteristics

A total of 349 patients were enrolled in the study: 101 patients attending the Department of Emergency Medicine of Azienda Ospedaliera Sant’Andrea, rome, in the first trial, 248 attending the outpatient clinic at Istituto Dermopatico dell’Immacolata, in the second trial. Of the patients enrolled in the first trial, antibiotic susceptibility testing was performed with both methods for 13 positive culture patients; among these, 7 (54%) were admitted with an ongoing antibiotic therapy (last antibiotic uptake within 12 hours before urine sampling) and the most frequently used antibiotics were metronidazole (43%), ciprofloxacin (29%), cefepime (14%), piperacillin-tazobactam (14%) and vancomycin (14%), used in combination only in one case. Of the patients enrolled in the second trial antibiotic susceptibility testing was performed with both methods for 84 culture-positive patients. Among these only 1 patient was admitted with an ongoing antibiotic therapy with ciprofloxacin (Table 1).

**Table 1:** Patients’ characteristics.

	Sex		Mean age	Catheterized	Antibiotic therapy
	F	M			
S. Andrea (n = 13)	11 (85%)	2 (15%)	78	12 (92%)	7 (54%)
IDI (n = 84)	61 (73%)	23 (27%)	71	6 (7%)	1 (1,2%)

### Hospital antibiotic susceptibility testing results

In the first clinical trial, at Azienda Ospedaliera Sant’Andrea, Rome, antibiotic susceptibility testing was routinely performed by the hospital laboratory for 13 positive urine cultures, and results were provided after 48–96 hours. Among the 7 patients undergoing antibiotic therapy (54%), AST results confirmed that 53% of the infecting microorganisms were resistant to previously administered antibiotics while only 23.5% were susceptible. In 23.5% of the cases the antibiotics used in therapy were not tested in AST.

In the second clinical trial, at Istituto Dermopatico dell’Immacolata, Rome, antibiotic susceptibility testing was performed by hospital laboratory for 84 positive urine cultures, and results were provided after 48 hours. Among these patients, only 1 was undergoing antibiotic therapy.

### MBS AST POCT antibiotic susceptibility test results

In the first clinical trial analyses with the MBS AST POCT were performed on all patients admitted in the ED immediately after urine collection. Given the small patient pool in this first trial, following data should be considered a proof of concept for the possible application of the MBS AST POCT for the preliminary susceptibility profiling of uroflora in an easier though reliable way, meaning directly from urine samples. The tested antibiotics were amoxicillin-clavulanic acid and levofloxacin.

Considering levofloxacin, in 9 cases color change occurred in the reaction vial containing the antibiotic, meaning the infecting bacteria resulted resistant (69%), while in 4 cases no color change occurred in the antibiotic supplemented reaction vial, and infecting bacteria were recorded as susceptible (31%). Considering amoxicillin-clavulanic acid, 11 samples resulted resistance (85%), while 2 samples susceptibility (15%). Agreement was reached for 12 out of 13 samples analyzed (92%), as all MBS AST POCT results were in agreement with those of hospital laboratory, except one. Given such discordance, this sample underwent verification analysis.

In the second clinical trial analyses were performed only on culture positive samples. This permitted to expand the pool of tested patients, allowing a more solid statistical analysis and a deeper understanding of MBS AST POCT potential. The tested antibiotics were amoxicillin-clavulanic acid, ciprofloxacin and trimethoprim-sulfamethoxazole: 57 samples were screened for susceptibility to amoxicillin-clavulanic acid, 80 to ciprofloxacin and 72 to trimethoprim-sulfamethoxazole respectively.

Considering amoxicillin-clavulanic acid, 36 samples resulted resistant (63%) and 21 (37%) susceptible; for ciprofloxacin results were respectively 34 (42.5%) resistant and 46 (57.5%) susceptible; for trimethoprim-sulfamethoxazole 27 samples were resistant (37.5%) and 45 susceptible (62.5%). Agreement was reached for a total of 193 samples out of 209 (92%), with 16 results (8%) showing discordance between the AST-POCT and the reference method. Following operating protocol, such samples underwent verification analysis.

**Fig 1:**
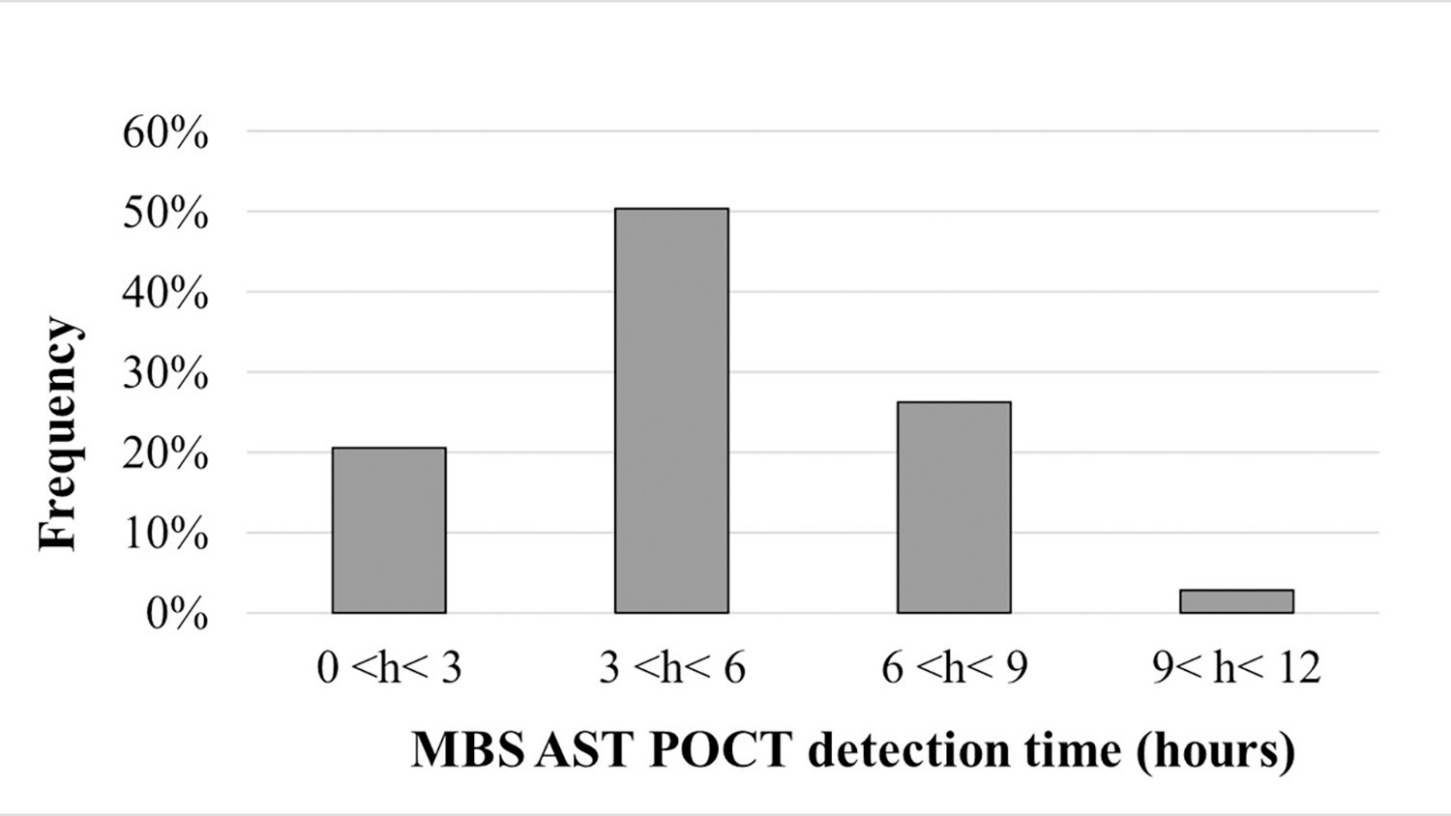
Detection time of resistant bacteria with MBS AST POCT. Histograms show detection frequencies (%) of MBS AST POCT resistant results at different time intervals.

All MBS AST POCT results were obtained within 12 hours. In particular, the average detection time to asses resistance to antibiotics was of 5 hours and results were obtained in less than 6 hours in 71% of cases (Fig 1).

This represents an important issue to consider when looking at the overall performance of the MBS method for diagnosis and management of UTI.

Previous studies, focused on the evaluation of the MBS POCT accuracy in performing bacterial load assessment for UTI detection, demonstrated that the MBS POCT was able to detect the presence of significant bacteriuria in maximum 5 hours [33, 34].

Taken together these results confirm the potential of the method: its adoption in routine clinical practice could provide, in short time, important information to the medical staff that, once the presence of UTI has been confirmed, could exclude ineffective antibiotics, and perform an evidence-based choice among treatment options, ultimately leading to more effective treatment and faster resolution. The fast response of the method could indeed reduce the adoption of empirical treatment and avoid the misuse and overuse of antibiotics, considered the major problem contributing to the emergence of resistant bacteria.

Moreover, its use in combination with other rapidly detected biomarkers as procalcitonin, high levels of which have been positively correlated to culture positive patients [35, 36], especially in case of urosepsis [37, 38], could represent an important screening for the identification and effective management of high-risk patients.

### Ex-post verification analysis

During the first trial, discordance between the results obtained with reference method and MBS AST POCT was observed for 1 patient out of 13 patients analyzed for both levofloxacin and amoxicillin-clavulanic acid. In this case, with the MBS AST POCT, the infecting bacteria resulted resistant to both the tested antibiotic, whereas the hospital report testified such bacteria as susceptible. Ex-post verification was thus carried out on frozen urine samples. The MBS AST POCT results were coherent with those obtained previously, confirming the presence of bacteria, resistant to both of the tested antibiotics. The Kirby-Bauer diffusion disk test showed the presence of two distinct bacterial strains displaying different susceptibility to both antibiotics, one being susceptible and one resistant. Because of the presence of a resistant strain and the ultimate ineffectiveness of the antibiotic in preventing bacteria multiplication in urine, the sample was considered resistant.

In the second trial, discordant AST results were obtained for 16 patients, samples of which underwent verification analysis according to the previously described operating protocol. Of these, 9 discordant results were obtained for amoxicillin-clavulanic acid. After ex-post verification, agreement between MBS AST POCT and reference method’s results was achieved for 5 samples, confirming MBS AST POCT initial results, and such data were then used for statistical analysis; in the 4 remaining cases, discordance between results persisted, thus MBS AST POCT results were included into statistical analysis as either false positive or false negative results. Considering ciprofloxacin, 3 discordant results were obtained, but verification analysis confirmed agreement for all samples, which led to complete agreement between MBS AST POCT and reference method’s results. Lastly, for trimethoprim-sulfamethoxazole, discordant results between methods were obtained for 4 samples. After ex-post verification, agreement was achieved for 2 samples (3%), while for the other 2 samples no agreement was obtained (3%), so these results were included into statistical analysis either as false positive or false negative.

The overall performance of the MBS AST POCT is showed in Tables 2 and 3. In detail, 100% accuracy was reached in the first trial for all antibiotics tested, though considering a quite narrow patient pool; 93%, 100% and 97% accuracy were reached respectively for amoxicillin-clavulanic acid, ciprofloxacin and trimethoprim-sulfamethoxazole in the second trial, considering a larger patient pool. Positive predictive value (PPV) and negative predictive value (NPV) are considered respectively as the proportions of resistant (positive) and susceptible (negative) results, that are true resistant and true susceptible results.

**Table 2:** Antibiotic susceptibility test results obtained with both MBS AST POCT.

Amoxicillin-clavulanic acid		Reference method			
	MBS AST POCT		Resistant	Susceptible	Total
		Resistant/Positive	42	2	44
		Susceptible/Negative	2	24	26
		Total	44	26	70
		Reference method			
Levofloxacin	MBS AST POCT		Resistant	Susceptible	Total
		Resistant/Positive	11	0	11
		Susceptible/Negative	0	2	2
		Total	11	2	13
Ciprofloxacin		Reference method			
	MBS AST POCT		Resistant	Susceptible	Total
		Resistant/Positive	35	0	35
		Susceptible/Negative	0	45	45
		Total	34	45	80
Trimethoprim-sulfamethoxazole		Reference method			
	MBS AST POCT		Resistant	Susceptible	Total
		Resistant/Positive	26	1	27
		Susceptible/Negative	1	44	45
		Total	27	45	72

The Antibiotic Susceptibility Test results obtained with MBS AST POCT were compared to those of the reference method, for the four antibiotics of interest. Data considered for analysis have been gathered following ex-post verification in case of discordant results.

**Table 3:** Performance of MBS POCT antibiotic susceptibility test.

	Accuracy (%)	Sensitivity (%)	Specificity (%)	PPV (%)	NPV (%)
Amoxicillin-clavulanic acid *n = 70* (13+57) *	94	95	92	95	92
Levofloxacin *n = 13*	100	100	100	100	100
Ciprofloxacin *n = 80*	100	100	100	100	100
Trimethoprim-sulfamethoxazole *n = 72*	97	96	98	96	98

The performance of MBS POCT Antibiotic Susceptibility Test was compared with reference method’s antibiogram’s for the antibiotics of interest, considering the patients enrolled in both studies. Accuracy, sensitivity, specificity, Positive Predictive Value (PPV) and Negative Predictive Value (NPV) have been calculated according to Friedman et al [39].

*(n_1_ = patients enrolled in the first study (S. Andrea) + n_2_ patients enrolled in the second study (Istituto Dermopatico dell’Immacolata).

A key issue brought to light by ex post verification analyses is the bias associated with selection of bacterial colonies for antibiogram profiling. Indeed, in case of polymicrobial infections, more than one bacterial strain is found in urine samples participating in the infection, possibly displaying different susceptibility to antibiotics. After plating, usually, only few colonies undergo further analysis, giving clinicians incomplete information about uropathogens resistance profile. It is not infrequent that prescribed therapy results ineffective. Antibiotic susceptibility assessment using the MBS AST POCT relies on culture, in the presence of antibiotics, of urine sample itself, meaning no colony selection occurs, taking into account all bacterial strains within the sample. In this way, the MBS AST POCT evaluates the antibiotic susceptibility of the overall bacterial pool, avoiding operator’s subjective interpretation of results.

### Impact of sample freezing on MBS AST-POCT results

Analyses on frozen samples were performed only on culture-positive samples, i.e., on samples for which urine culture and AST results were made available by the hospital’s laboratory. Analyses were performed after 2, 5 and 7 days. Results, in terms of susceptibility/resistance to the tested antibiotics, were always in agreement with those obtained on fresh samples (data not shown) meaning that freezing urine samples did not significantly affect the response of the MBS AST-POCT within a week. Despite the MBS AST-POCT has been conceived as near-patient testing, this result has been very important to optimize operating procedures, namely helping to exclude samples unsuitable for AST POCT analysis (e.g., evident macrohematuria) and to investigate and clarify cases in which discordant results were obtained from reference method and MBS AST POCT analysis [33, 34]. Given the difficulty to perform analyses on all patients in the first clinical trial, demonstrating that sample freezing did not impact on MBS AST POCT results, allowed to expand the pool of patients tested and provide an alternative procedure to test only culture-positive samples, that could prove advantageous in low resource settings or in clinical settings characterized by a high prevalence of culture negative patients.

## Conclusions

In this paper we evaluated the performance of a new Point Of Care Test (POCT) for the rapid prediction of antimicrobial susceptibility of uropathogens. 349 patients were enrolled in two open-label, monocentric, non-interventional clinical trials in partnership with an Emergency Medicine ward and the Day Hospital of two large healthcare facilities in Rome, Italy. The pool of patients and antibiotic tested was contained, therefore the study can be considered as a preliminary investigation that has, however, provided promising results.

Overall, the MBS AST POCT displayed high accuracy (greater than 90%), and PPV (greater than 95%) with all the tested antibiotics, and provided results in less time compared to traditional methods, especially for resistant bacteria, that were detected within 12 hours from urine collection.

Based on these evidences, the use of such a POCT in routine clinical practice could have an important impact, allowing an accurate evaluation of susceptibility/resistance of the infecting bacteria to selected antibiotics, though reducing analytical time and labor and diminishing analytical and management costs. Yielding results within a working day would indeed allow clinicians to promptly treat patients without the need of empiric antibiotic therapy, thus ultimately, helping to counteract the rise of antibiotic resistance.

The device provides important information about the efficacy of antibiotics of interest directly testing urine samples and thus taking into account the overall urinary bacterial population, therefore preventing important data loss. The method itself does not provide the identification of pathogenic agents, however this can be easily achieved immediately after the analysis, using positive vials as bacterial cultures, ready to be further analyzed with standard techniques.

Moreover, the MBS AST POCT could prove useful in the assessment of local antimicrobial susceptibility patterns providing a tool for the rapid screening of urine samples and allowing to build an antibiotic susceptibility database within the hospital even in low-resource settings.
