# Expression of Concern: A new point-of-care test for the rapid antimicrobial susceptibility assessment of uropathogens

**DOI:** 10.1371/journal.pone.0362446

**Published:** 2026-11-11

**Authors:** The PLOS One Editors

After this article [1] was published, concerns were raised regarding undisclosed potential competing interests, clinical ethics, methods, and similarity with previously published articles by the same group.

Regarding the competing interests:

Based on information gathered in editorial follow-up, the updated Competing Interests statement is: GA is inventor of the Micro Biological Survey (MBS) method, a colorimetric method for counting bacterial load. This article [1] evaluates the validity of the MBS method for assessing bacterial load in urine. GA is listed as an inventor of patent EP2041297 (for a colorimetric bacterial load detection method); MBS SRL, a spin-off company of Roma Tre University that commercializes products based on MBS method, is the current assignee and owned the patent at the time of publication of [1]. GA supervises technical activities of MBS srl on behalf of Roma Tre University, but does not receive financial support from MBS srl. At the time of submission of this article [1], GA was a shareholder of MBS Diagnostics Limited, which developed an MBS-based test for use in urinary tract infections; MBS Diagnostics Limited is the assignee of patent EP3433606A1.

The corresponding author GA disagrees that the above information falls within the scope of the interests that must be declared for this article per the journal’s editorial policy.

- Regarding the study design and methodology: A concern was raised that for the ex-post analysis of samples with discordant results, the method for re-testing did not adequately address potential sample contamination. The information reported in [1] suggests that for re-testing of discordant results, the same urine sample was directly cultured and tested using the Kirby-Bauer disc diffusion (agar plate) method, and in a mixed population of resistant (R) and sensitive (S) strains, the whole sample was classified as resistant.
- [1] does not appear to include:
  ◦ Inclusion and exclusion criteria.
  ◦ A clear description of urine collection, sample processing, and analysis.
  ◦ The types of pathogens found.
  ◦ A discussion of potential limitations of certain antibiotics being tested in specific hospitals or patients.

The corresponding author declined to provide a response to the methodology concerns.

A member of the *PLOS One* Editorial Board stated that re-testing frozen samples rather than validating results using freshly collected samples and classifying whole samples as resistant when the agar plate shows a mixed population of resistant (R) and sensitive (S) strains is concerning because if the resistant strain was a contaminant introduced during sample collection and not the actual cause of infection, the patient could be treated incorrectly. They advised that this should be discussed as a limitation of the proposed method, and that it should be explicitly stated that this method must be validated with other cohorts before it can be recommended as a replacement for current standard patient care practices.

Regarding the clinical ethics and overlap with prior studies:

- [1] appears closely related to a previously published article by the same group [2], which was cited as Reference 34 in [1], but the extent of any overlap between the two articles in patient population, urine samples, and experimental procedures are not reported and discussed.
- Studies reported in [1] and [2] were carried out under the same approval from Ethical Committee of Istituto Dermopatico dell’Immacolata; studies reported in [1] and [3] were carried out under the same approval from Ethical Committee of the Azienda Ospedaliera Sant’Andrea. There is an error in the Ethical approval section in [1]: the Ethical Committee of Azienda Ospedaliera Sant’Andrea approved the clinical study on 2013/01/14 (not on 2019/01/14 as stated in [1]). It is not clear whether the study carried out at the Azienda Ospedaliera Sant’Andrea reported in [1] was covered by the approval issued in 2013.

The corresponding author stated that [2] contains clinical data concerning the diagnosis of urinary tract infections whereas [1] contains clinical data concerning how to choose the right antibiotic for the treatment of a urinary tract infection. They did not comment on whether the two articles report work using the same patient population and urine samples; however, the *PLOS One* Editors note that both articles report results from MBS-POCT testing of urine samples collected from the same number of patients enrolled in two open-label, monocentric, non-interventional clinical trials in collaboration with the same two hospitals. The *PLOS One* Editors therefore consider there is an unresolved concern about inadequate reporting about the independence of the two studies.

The corresponding author stated that the studies reported in [1–3] were conducted under the same initial ethical approvals, noting that the clinical studies lasted several years and this is allowed under the ethical approvals from Istituto Dermopatico dell’Immacolata and from Ethical Committee of the Azienda Ospedaliera Sant’Andrea. They provided copies of the ethical approval documents for editorial review.

The *PLOS One* Editors note that the document issued by the Ethical Committee of the Azienda Ospedaliera Sant’Andrea gave approval for participation of 150 patients from the Emergency Department of the hospital. The report of the pilot study [3] states that 122 patients were enrolled at this location in the period November 2013 – July 2014, while the later study [1] reports enrollment of 101 patients between November 2015 and December 2017; it appears that the total patient number enrolled at the Emergency Department of Azienda Ospedaliera Sant’Andrea exceeds the number stated in the ethical approval document. The *PLOS One* Editors were unable to obtain further information about ethical oversight from the institution. On the basis of the available documentation, it is not clear whether the later study at this location was appropriately covered by an extension of the original ethical approval or received the appropriate ethical oversight. The corresponding author stated that the approval granted by the Ethical Committee of the Azienda Ospedaliera Sant'Andrea did not set a maximum number of patients, nor a time limit for carrying out the research, and that the figure of 150 patients refers to the sample size estimated in the original protocol, not to a limit set by the Ethical Committee. They also stated that [1] and [3] were both conducted within the scope of this approval and in full compliance with its conditions and that no renewal or extension of the approval was required from Sant'Andrea for the enrolment of all patients.

The *PLOS One* Editors issue this Expression of Concern to inform readers of the unresolved concerns regarding methodology, reporting, and ethical oversight listed above, as well as the information about undeclared potential competing interests.

The *PLOS One* Editors also note the following additional information, which did not contribute to the decision to issue an Expression of Concern: MBS Diagnostics Limited appears to have been dissolved in March 2023 and therefore the test reported in [1] may no longer be available for replication of the results in [1].
